# Supplementary material for: Linker Length Drives Heterogeneity of Multivalent Complexes of Hub Protein LC8 and Transcription Factor ASCIZ
Source: Biomolecules. 2023 Feb 21;13(3):404. doi: 10.3390/biom13030404 (PMC10046861; doi:10.3390/biom13030404)
Supplement: Supplementary file 1 [file biomolecules-13-00404-s001.zip › biomolecules-2197895-supplementary.pdf]

Table S1. Thermodynamic parameter fits of the QT-LC8 interactions.

Construct	N		Overall K_d (μM)	Overall ΔH (kcal/mol)	Overall TΔS (kcal/mol)	Overall ΔG (kcal/mol)
QT 2-4	2.7 ± 0.02		4.8 ± 0.2	-13.9 ± 0.2	-6.6 ± 0.2	-7.3 ± 0.03
QT 2	1.0 ± 0.01		9.3 ± 0.3	-10.7 ± 0.1	-3.9 ± 0.1	-6.9 ± 0.02
QT 3	1.2 ± 0.02		36 ± 2	-8.4 ± 0.3	-2.3 ± 0.3	-6.1 ± 0.03
QT 4	1.0 ± 0.01		15 ± 1	-16.1 ± 0.3	-9.5 ± 0.3	-6.6 ± 0.03
QT 2,3	1.9 ± 0.02		6.3 ± 0.3	-9.2 ± 0.1	-2.1 ± 0.1	-7.1 ± 0.03
QT 3,4	1.1 ± 0.02		4.4 ± 0.4	-12.3 ± 0.4	-5.0 ± 0.4	-7.3 ± 0.06
QT 2,4	1.4 ± 0.01		1.8 ± 0.1	-13.2 ± 0.2	-5.4 ± 0.	-7.8 ± 0.04
QT2-4 sequential sites	QT2		6.2 ± 1.1	-15.9 ± 0.9	-8.9 ± 0.9	-7.1 ± 0.1
	QT4		7.6 ± 1.2	-18.2 ± 2.0	-11.2 ± 2.0	-7.0 ± 0.1
	QT3		27 ± 4	-1.4 ± 2.2	4.9 ± 2.2	-6.2 ± 0.1
QT2,3 sequential sites	QT2		0.9 ± 0.1	-7.9 ± 0.1	0.4 ± 0.1	-8.3 ± 0.07
	QT3		15 ± 1	-10.1 ± 0.1	-3.5 ± 0.2	-6.6 ± 0.02
QT3,4 sequential sites	QT4		2.2 ± 0.2	-11.5 ± 0.3	-3.8 ± 0.3	-7.7 ± 0.1
	QT3		40 ± 9	-2.4 ± 0.6	3.6 ± 0.7	-6.0 ± 0.2
QT2,4 sequential sites	QT2		0.11 ± 0.04	-12.4 ± 0.3	-2.9 ± 0.5	-9.5 ± 0.3
	QT4		3.2 ± 0.8	-5.6 ± 0.4	1.9 ± 0.4	-7.5 ± 0.2
QT2,3 two-sets of sites	QT2	1.4 ± 0.2	1.3 ± 0.5	-8.0 ± 0.2	0.02 ± 0.4	-8.0 ± 0.3
	QT3	1.1 ± 0.8	18.9 ± 4.1	-6.7 ± 7.3	-0.2 ± 7.3	-6.4 ± 0.2
QT3,4 two-sets of sites	QT4	0.8 ± 0.2	1.9 ± 1.4	-10.9 ± 1.3	-3.1 ± 2.1	-7.8 ± 1.6
	QT3	0.2 ± 3.5	25.7 ± 13.5	-30.7 ± 580	-24.4 ± 580	-6.3 ± 0.7
QT2,4 two-sets of sites	QT2	1.2 ± 0.1	0.5 ± 0.2	-12.5 ± 0.2	-3.8 ± 0.5	-8.6 ± 0.4
	QT4	0.6 ± 0.6	10.6 ± 3.1	-5.7 ± 7.3	1.1 ± 7.3	-6.8 ± 0.2

(Top) Simple origin fits to the "Single set of sites" model which assumes that each binding site present in the protein is equivalent. (Middle) Origin fits to the "Sequential binding site" model which assumes that binding events must occur in a particular order. (Bottom) Origin fits to the "two-sets of sites" model which assumes there are two distinct types of binding sites in a protein.

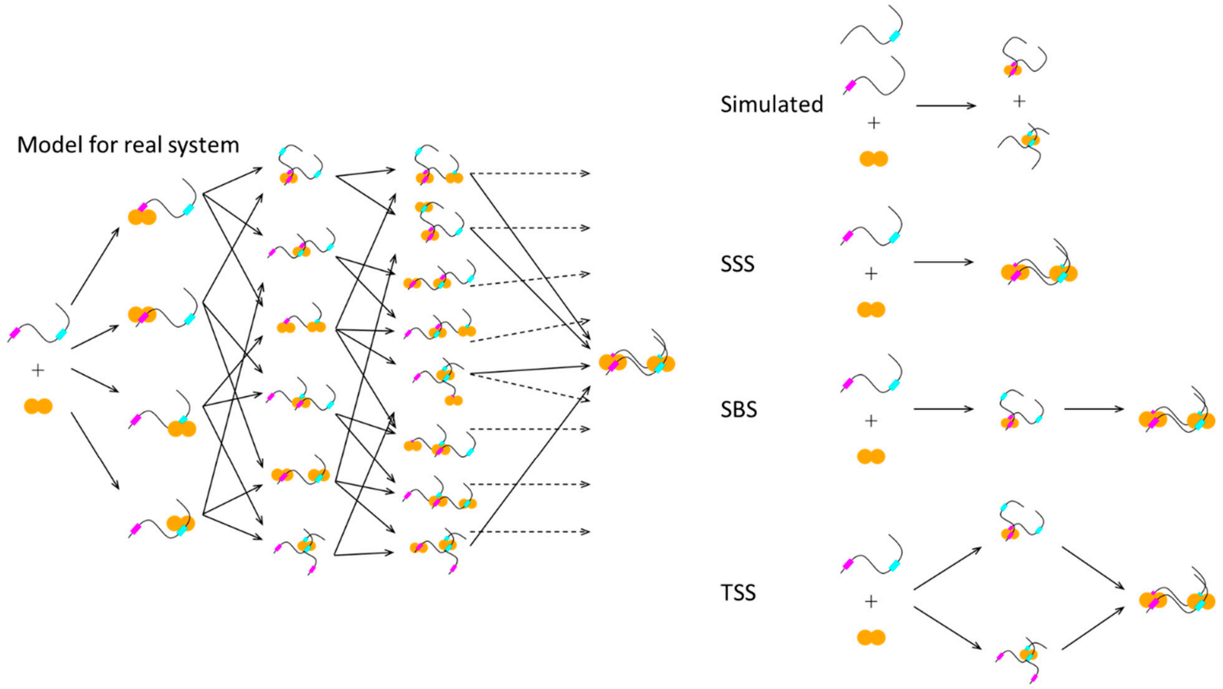

Figure S1) Algorithm Models. Model of what is happening in the ITC titrations of the double-site variants compared to the models used by the various algorithms discussed in this paper to try to assess these systems.

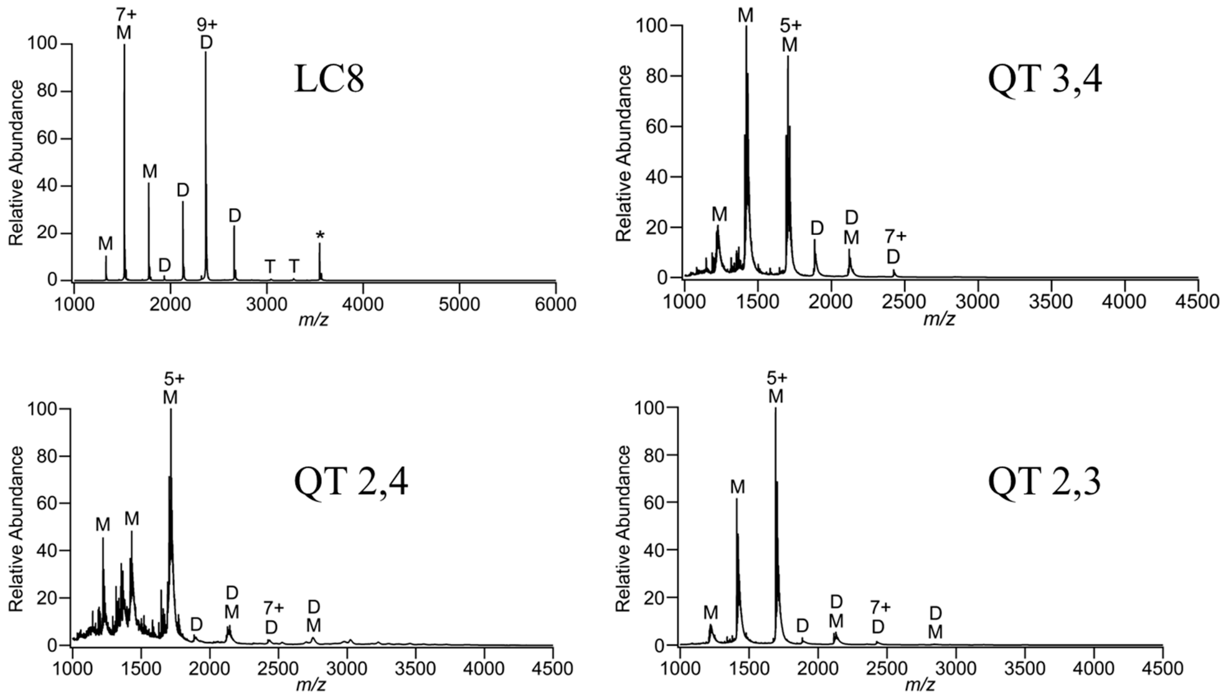

Figure S2) Mass spectra of each of the individual species analyzed. * $p \leq 0.05$.

Table S2. Observed versus expected masses for individual proteins and complexes

Protein	Charge states	Sequence mass (Da)	Measured mass (average ± s.d., Da)
LC8	6-10+	10639.11	10638.75 ± 0.04
QT2,3	3-7+	8475.26	8452.77 ± 0.07
QT2,4	4-7+	8472.20	8510 ± 1
QT3,4	3-7+	8475.26	8452.47 ± 0.27
**Species/complex**	**Charge states**	**Expected mass (Da)**	**Measured mass (average ± s.d., Da)**
LC8 monomer	6-9+	10638.75 ± 0.04	10638.82 ± 0.04
LC8 dimer	8-11+	21277.51 ± 0.07	21278.3 ± 0.5
LC8 tetramer	11-14+	42555.01 ± 0.15	42558 ± 2
QT3,4 monomer	5-7+	8452.5 ± 0.3	8452.4 ± 0.5
QT3,4 monomer + LC8 dimer	9-13+	29729.98 ± 0.28	29732.52 ± 0.21
QT3,4 monomer + 2 LC8 dimers	13-16+	51007.49 ± 0.31	51013 ± 7
QT3,4 dimer + 2 LC8 dimers	13-20+	59460.0 ± 0.6	59464 ± 16
QT3,4 dimer + 3 LC8 dimers	16-20+	80737.5 ± 0.6	80766 ± 20
QT2,4 monomer	5-7+	8510 ± 1	8508 ± 6
QT2,4 monomer + LC8 dimer	9-13+	29788 ± 1	29788.1 ± 0.2
QT2,4 monomer + 2 LC8 dimers	15-18+	51065 ± 1	51073 ± 4
QT2,4 dimer + 2 LC8 dimers	14-21+	59576 ± 2	59576 ± 21
QT2,4 dimer + 3 LC8 dimers	16-20+	80853 ± 2	80854 ± 11
QT2,3 monomer	5-7+	8452.77 ± 0.07	8452.63 ± 0.3
QT2,3 monomer + LC8 dimer	10-12+	29730.28 ± 0.10	29732.01 ± 0.33
QT2,3 monomer + 2 LC8 dimers	12-16+	51007.78 ± 0.16	51036 ± 22
QT2,3 dimer + 2 LC8 dimers	13-20+	59460.56 ± 0.20	59510 ± 31
QT2,3 dimer + 3 LC8 dimers	15-20+	80738.06 ± 0.26	80804 ± 54
